# Multimodal Optical Diagnostics of the Microhaemodynamics in Upper and Lower Limbs

**DOI:** 10.3389/fphys.2019.00416

**Published:** 2019-04-16

**Authors:** Angelina I. Zherebtsova, Viktor V. Dremin, Irina N. Makovik, Evgeny A. Zherebtsov, Andrey V. Dunaev, Alexey Goltsov, Sergei G. Sokolovski, Edik U. Rafailov

**Affiliations:** ^1^Research and Development Center of Biomedical Photonics, Orel State University, Oryol, Russia; ^2^Optoelectronics and Measurement Techniques Unit, University of Oulu, Oulu, Finland; ^3^Optoelectronics and Biomedical Photonics Group, Aston Institute of Photonic Technologies, School of Engineering and Applied Science, Aston University, Birmingham, United Kingdom; ^4^School of Applied Sciences, Abertay University, Dundee, United Kingdom; ^5^International Center of Critical Technologies in Medicine, Saratov State University, Saratov, Russia

**Keywords:** laser Doppler flowmetry, tissue reflectance oximetry, pulse oximetry, fluorescence spectroscopy, skin thermometry, rheumatic diseases, diabetes mellitus

## Abstract

The introduction of optical non-invasive diagnostic methods into clinical practice can substantially advance in the detection of early microcirculatory disorders in patients with different diseases. This paper is devoted to the development and application of the optical non-invasive diagnostic approach for the detection and evaluation of the severity of microcirculatory and metabolic disorders in rheumatic diseases and diabetes mellitus. The proposed methods include the joint use of laser Doppler flowmetry, absorption spectroscopy and fluorescence spectroscopy in combination with functional tests. This technique showed the high diagnostic importance for the detection of disturbances in peripheral microhaemodynamics. These methods have been successfully tested as additional diagnostic techniques in the field of rheumatology and endocrinology. The sensitivity and specificity of the proposed diagnostic procedures have been evaluated.

## Introduction

Blood microcirculation plays an important role in the transport of nutrients, oxygen, hormones and the release of metabolic products. In the last decade, there has been a steady increase in the interest of researchers in the problems of microcirculatory disorders in patients with rheumatological and endocrinological diseases. This is due to the significant role of microcirculation in the pathogenesis of such diseases (Avouac et al., 2011; Sena et al., 2013; Fuchs et al., 2017).

The medical, social and economic significance of rheumatic and endocrinological diseases is determined primarily by their prevalence, as well as the development of complications in the majority of patients, which significantly reduce the duration and quality of life, up to disability. According to European League Against Rheumatism (EULAR) statistics, more than 22% of the population already has or had previously rheumatic diseases (Smolen et al., 2017). At the same time, according to the International Diabetes Federation (IDF), by 2017 the prevalence of diabetes mellitus in the world has reached 8.8% among the adult population (Karuranga et al., 2017).

Rheumatic diseases lead to morphological disturbances of the microcirculatory bed, which include the rarefaction of the capillary network, the asymmetry of the capillaries, and the appearance of megacapillaries (up to 50 μm in diameter) (Gutierrez et al., 2010). Chronic hyperglycemia and insulin resistance in diabetes mellitus cause increased vascular permeability, disruption of autoregulation of blood flow and vascular tone (Avogaro et al., 2011; Kolluru et al., 2012), leading to the structural and functional changes in capillaries and arterioles (Schramm et al., 2006).

In the absence of adequate therapy, peripheral blood flow disorders in limbs can lead to painful ulceration, gangrene, and the development of cardiovascular diseases. Previously published studies have shown that the risk of developing cardiovascular disease in patients with rheumatic diseases is comparable to that in patients with type 2 diabetes (Peters et al., 2010; Kerekes et al., 2012).

The earliest, usually reversible manifestation of these diseases is the development of microcirculatory dysfunction (Vanhoutte et al., 2015; Fuchs et al., 2017) due to endothelial damage, excessive expression of certain adhesion molecules and other factors. At present, several invasive and non-invasive methods are used in the clinical practice of endocrinologists and rheumatologists, including colorimetric duplex dopplerography, angiography, computed tomography, magnetic resonance angiography, transcutaneous oximetry (*T_cp_O*_2_), etc. (Daly Susan and Leahy Martin, 2012).

The methods of optical diagnostics are promising for the study of early microcirculatory disorders in patients with rheumatic diseases and diabetes. These methods have several advantages: painless procedures, quick results, lack of expensive consumables, minimal impact on the object and its properties. This article presents an overview on recent advances in optical non-invasive diagnostics of the peripheral hemodynamics of the upper and lower limbs in rheumatological and endocrinological profile patients. Among these are laser Doppler flowmetry (LDF), tissue reflectance oximetry (TRO), pulse oximetry (PO) and fluorescent spectroscopy (FS).

The laser Doppler flowmetry method allows for investigating the blood flow in the microcirculatory bed *in vivo*. The method is based on probing the tissue with laser radiation and analyzing back reflected from the tissue radiation partially scattered from moving red blood cells (Bonner and Nossal, 1990; Krupatkin and Sidorov, 2013).

The tissue reflectance oximetry method provides information about the tissue oxygen saturation (*S*_t_*O*_2_) of the examined biological tissue microcirculation and allows for calculation of the relative blood volume (*V*_b_) in the surface layers of soft tissues (skin, mucous membranes). This technology is based on the ability of oxygenated and deoxygenated hemoglobin to absorb light in the red and near infrared range (Casavola et al., 1999; Wallace et al., 2009).

Mechanisms that cause abnormal microcirculation in pathological processes can be quantified by use of wavelet transform for the records of LDF and TRO signals. The amplitude-frequency analysis of oscillations in skin blood flow allows distinguishing five frequency ranges corresponding to metabolic (endothelial) (Kvandal et al., 2003), neurogenic, myogenic (Söderström et al., 2003; Krupatkin, 2006), respiratory and cardiac (Stefanovska et al., 1999; Krupatkin, 2008) activity.

Optical diagnostics enables not only evaluation of blood flow parameters but registration of associated biochemical changes in the living tissue under study. The fluorescence spectroscopy is one of such methods allowing for registration metabolic changes *in vivo*. As an example, the method has promises to be applied in diagnostics of the diabetes mellitus complications. An important indicator of the viability of tissues is the mitochondrial function. By the parameters of the respiratory chain, one can speak of normal or pathological activity of the cells, diagnose the state of tissue ischemia. One of the estimates of the mitochondrial function is the ratio of coenzymes NADH and FAD, which can be calculated from the intensity of their fluorescence (Bartolomé and Abramov, 2015). In addition, in recent years, it has been found that the long-term effects of pathogenic factors such as hyperglycemia and oxidative stress in diabetes mellitus (Tan et al., 2002) and chronic inflammatory diseases (Murdaca et al., 2012) can lead to increased glycation of proteins and accumulation of advanced glycation end products (AGEs), which affect the properties of collagen and other structural proteins of the capillary membrane and skin (Gkogkolou and Böhm, 2012). These changes can be quantified by the intrinsic fluorescence of the pentosidine residues formed during glycation of collagen (Sell et al., 1991; Takahashi et al., 1995).

To increase the reliability of the results obtained by the optical spectroscopy methods, an assessment of the measured parameters changes is carried out with functional tests. Occlusion, heat, cold, respiratory, orthostatic, electrostimulation et al. tests are currently used as an act of provocation. Functional tests allow for revealing latent hemodynamic disorders and adaptive reserves of the microcirculation system.

The recent studies show that multiparametric approach in the form of joint measurements by various optical methods supplemented by functional tests can give complex information about the functional state of the microcirculation (Goltsov et al., 2017; Zherebtsov et al., 2017; Zherebtsova et al., 2017).

The aim of this work is to generalize the previously obtained results (Dremin et al., 2017; Mizeva et al., 2017, 2018; Zherebtsov et al., 2017; Zherebtsova et al., 2017) and to assess the possibilities of using optical non-invasive methods in studying the microcirculatory bed of patients with disorders in peripheral microhemodynamics.

The article provides a review of three methods of combined use of optical non-invasive technologies for the diagnostics of blood microcirculation and metabolic disorders. The main distinctive feature of all described methods is the use of functional tests, which greatly enhanced the diagnostic capabilities of the methods (Herrick and Clark, 1998).

The first two experimental studies demonstrate the possibility of using optical non-invasive methods and cutaneous temperature for detection violations of peripheral blood flow of the upper limbs in rheumatological profile patients. The cold water exposure, occlusion tests (OT) and cold pressor test (CPT) were selected as a provocative actions. Occlusion test is most commonly used functional test to investigate and assess microvascular function according to post occlusive reactive hyperemia (PORH) response (Roustit and Cracowski, 2012). A cold test is also often used for diagnostic purposes due to vasospasm is characterized by sporadic manifestation and influenced by trigger factors like cold exposure, emotional stress, physical exercise etc. (Terada et al., 2007; Ye and Griffin, 2016). The deliberate provocation of vasospasm allows one to increase the sensitivity of the diagnosis and to assess the severity of the pathological process.

The third experimental study estimates the potential of synchronous registering the blood flow parameters and the fluorescence of intrinsic tissue fluorophore with the purpose of diagnosing the stages of complications of lower limbs in diabetes mellitus patients. The local heating stimulation was chosen as test action on the blood microcirculation system. The local heating test allows the assessment of the local regulatory mechanisms of blood flow.

## Simultaneous Measurements of the Blood Perfusion and Skin Temperature for Functional Diagnostics of Intradermal Finger Vessels

### Materials and Methods

The aim of this study was evaluation of the combined use of the laser Doppler flowmetry and skin thermometry methods during the occlusion test to distinguish vasospastic disorders in hands (Zherebtsov et al., 2017). The experimental studies involved 27 healthy volunteers (HV) (mean age 23 ± 5 years) and 41 patients with rheumatic diseases (PRD) (mean age 56 ± 12 years) from the Rheumatology Department of the Orel Regional Clinical Hospital (Oryol, Russia). Subjects in the HV group did not have diagnosed diseases that are accompanied by secondary vasospastic syndrome, and did not have signs and symptoms which are indicative for primary vasospasm or predisposition to vasospasm [e.g., cold hands and feet, low blood pressure (Flammer et al., 2001)]. Presence of vasospasm of each tested subject in the PRD group was confirmed by the attending physician.

It is well known that microcirculatory abnormalities contribute to the pathogenesis and pathophysiology of numerous rheumatic diseases (Murray et al., 2004). Microvascular changes taking place in the early stages of such diseases may not manifest itself. In many cases, vascular problems can remain hidden. Such a high risk factor as aging can significantly contribute to the vascular state of the volunteers (Abularrage et al., 2005). For this reason, before carrying out measurements in the group of patients with diagnosed rheumatic diseases, we have tried to find a control group of people in their 50s without clear signs of vascular diseases. Nevertheless, due to the lack of confidence on absence of vascular disorders among the aged volunteers, the younger people who are more likely have no microcirculatory disorders were intentionally included in the control group.

**FIGURE 1:**
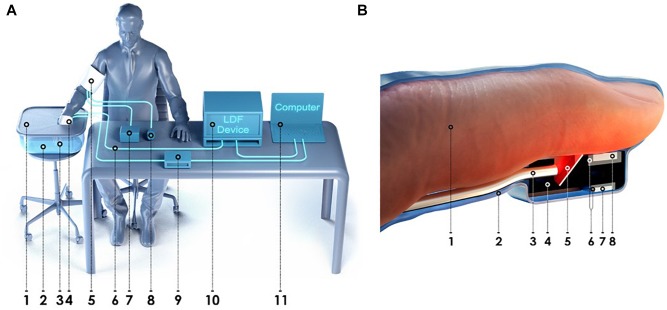
The experimental setup for simultaneous measurements of the blood perfusion and skin temperature **(A)**: 1 – heat-insulated cover; 2 – heat-insulated water bath; 3 – specially designed attachment for the LDF fiber probe; 4 – latex glove; 5 – arm cuff; 6 – LDF fiber probe; 7 – manometer; 8 – inflation bulb; 9 – thermometry device; 10 – LDF device; 11 – PC. Design features and location of the proposed attachment for the LDF fiber probe and thermal sensors **(B)**: 1 – third finger; 2 – latex glove; 3 – LDF fiber probe; 4 – housing of the attachment; 5 – mirror; 6 – thermal insulation; 7 – ambient temperature thermal sensor; 8 – skin temperature thermal sensor.

The laser analyser of blood microcirculation “LAKK-02” (SPE “LAZMA” Ltd., Russia) and a custom developed multi-channel thermometry device for low inertia thermometry were used for experimental measurements. The measurements of cutaneous temperature and the index of microcirculation were performed on the distal phalanx of the third finger of the right hand. The specially designed attachment was used for longitudinal arrangement of the LDF fiber probe. The experimental setup and design features and location of the proposed attachment is presented in Figure 1.

**FIGURE 2:**
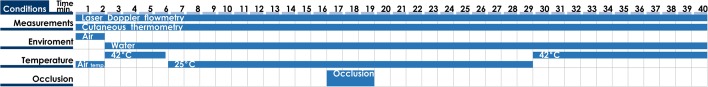
Time chart of the study protocol of simultaneous measurements of the blood perfusion and skin temperature.

The stage of heating of the hand at 42°C was applied for secure equal initial conditions of the experiment. This temperature ensures complete dilatation of vessels and the rate of blood flow returns to a normal level, even in patients with vasospastic disorders. The temperature of the main part of experiment was 25°C. It was selected from considerations of comfort for patients and maximal potentially possible dynamic range of the skin postocclusion temperature response. Occlusion test was performed on the upper arm using a sphygmomanometer air cuff with a pressure of 200–220 mmHg for 3 min. The experiment was conducted according to the study protocol is described in Figure 2.

**FIGURE 3:**
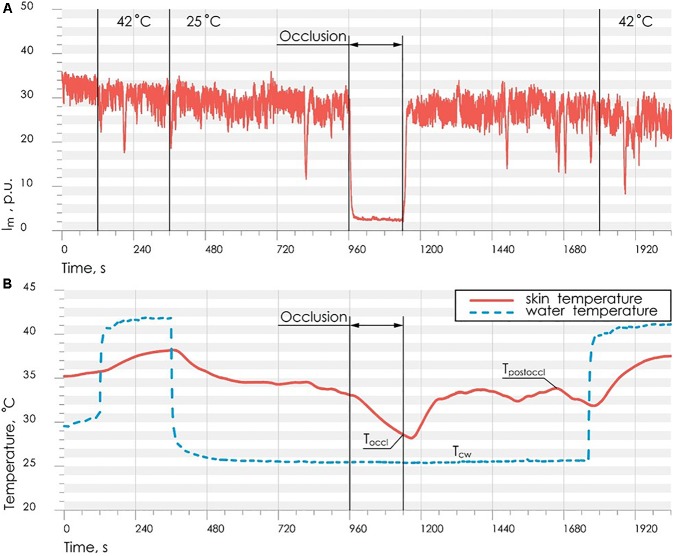
Representative record of the cutaneous blood perfusion **(A)** and skin temperature **(B)** during the study on the conditionally healthy volunteer.

All measurements were carried out mainly in the morning, 2–3 h after meal, in a state of mental and physical rest. The patient sat in such a way that the forearm of his right hand was 20 cm below the level of the heart (Figure 1A). The total duration of the experiment was not more than 40 min. Representative record of the cutaneous blood perfusion and temperature of the conditionally healthy volunteer is shown in Figure 3A,B, respectively.

### Data Analysis and Diagnostic Criteria

Based on the results of the study, it was revealed several characteristic types of the blood microcirculation system response to temperature and occlusion effects (Zherebtsova et al., 2015).

The first type of response is characterized by the absence of the spasm, that reflects in temperature and perfusion increased in post-occlusion period.

In the case of the second type of the response, the initial parameters of the thermoregulatory system are such that at a moderate cooling of fingers the system can rapidly switch to state with low temperature. However, the effect of vasodilation induced by the occlusion is higher than the vasoconstriction induced by cooling. Thus, the mechanism of endothelial function for patients with the second type of response remains active.

In the case of the third type of response, vasospasm tendency remains much high (a tendency to stay in the stationary point of low temperature) and the vasodilatory effect of the occlusion is minimal. Therefore, the vasodilation does not appear in this case against a background of the vasoconstriction of vessels for the present temperature level.

The analysis of the obtained results showed that for the reliable identification of the functional state of blood microcirculation in fingers it is advisable to use two parameters based on the type of response of the cutaneous temperature and the skin blood flow to the arterial occlusion at a low temperature.

For the assessment of the vasodilation effect on cutaneous temperature, it was proposed to estimate the ratio of the difference between the maximum temperature after occlusion and the temperature of cold water to the difference between the minimum cutaneous temperature during occlusion and the temperature of cold water. To account for the effect of biological tissue heat capacity, the obtained value was normalized to the volume of the distal phalanx of the finger. Thus, the index of temperature response (*ITR*, arb. un.), based on skin temperature measurements, was calculated by the following formula:

(1) $$ITR=\frac{1}{V}\frac{\left(T_{\text{PO}}-T_{\text{CW}}\right)}{\left(T_{\text{O}}-T_{\text{CW}}\right)}$$

where *T*_O_ – the minimum temperature of the biological tissue during occlusion period, °C; *T*_PO_ – the maximum temperature of the biological tissue during occlusion period, °C; *T*_CW_ – the temperature of cold water in the heat-insulated water bath; *V* – the volume of the distal phalanx of the test finger, calculated as the volume of semi-ellipsoid, cm^3^.

*ITR* allows assessing the extent of post-occlusion microvessels vasodilatation at artificially created low ambient temperature. Vasospasm can be described as a state when thermoregulatory system tends to the stable stationary point that is close to ambient temperature (“stationary point with low temperature”). Whereas in case of normality the thermoregulation is in a stationary point which is characterized by a temperature above the ambient temperature (“stationary point with high temperature”). Formation of the feedforward trigger system of switching between the stationary states is caused by the positive non-linear relationship between temperature and blood perfusion (Nakamura, 2011). Decrease in the temperature of the biological tissue leads to decrease in the blood perfusion, which in turn contributes to the further temperature reduction. The effect of vasoconstriction due to temperature falling can be compensated by vasodilation effect of occlusion (Dezfulian et al., 2017), which appears in normal conditions and characterizes the functional state of vascular endothelium in norm (Meredith et al., 1996). Decrease of index of the temperature response was observed in the PRD group.

However, the use of parameters from only cutaneous thermometry does not allow the relevant separation of the norm and presence of vasospastic disorders. In this connection, we suggest to use the composite diagnostic criteria, which includes both LDF and cutaneous thermometry parameters.

For the assessment of adaptation reserves in the blood microcirculation system and for the evaluation the process of skin blood flow restoration after occlusion, the most commonly used value is the percentage ratio between the maximum skin blood perfusion after occlusion and the average skin blood perfusion level in basal conditions. In these experiments, we used the parameter called blood flow reserve (*BFR*, %), calculated according to the formula:

(2) $$BFR=\frac{Im_{\max}}{Im_{\text{base}}}100\%$$

where *Im*_max_ – average index of blood microcirculation in the first 60 s after occlusion, PU; *Im*_base_ – average index of blood microcirculation during 60 s before occlusion, PU. Averaging of skin blood flow during 60 s is used to exclude the influence of motion artifacts on the results of calculations.

Post occlusive reactive hyperemia is used to investigate and assess microvascular function (endothelial function). Therefore, the *BFR* parameter characterizing the reaction of microvessels to arterial occlusion can serve as an effective tool for assessing microcirculatory disorders in rheumatic diseases.

In order to synthesize the decision rule for diagnosis, it is necessary to find a discriminant function that separates classes of the norm and the presence of vasospastic disorders using the *ITR* and *BFR* parameters.

**FIGURE 4:**
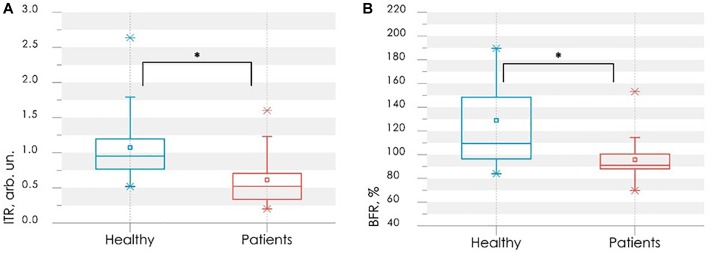
Comparison of parameters between control (blue bars) and rheumatic (red bars) groups: the index of temperature response ITR **(A)** and the blood flow reserve BFR **(B)**. In each box, the central line is the median of the group, while the edges are the 25th and 75th percentiles. Statistically significant differences between the comparing groups with ^∗^ is *p* < 0.005 (according to Mann–Whitney *U*-test).

Experimental studies have shown that PRD have lower values of the blood flow reserve, as well as a reduced index of temperature response after occlusion. There is a statistically significant difference of *BFR* and *ITR* parameters between values calculated for the HV and PRD. Comparison of these parameters between studied groups is shown in Figure 4.

The values of *BFR* and *ITR* have a two-dimensional normal distribution. The training sample size *n* = *n*_1_ + *n*_2_ = 68 is more than 20 times larger than the number of variables in the vector of informative parameters *m* = 2.

All these conditions enable us to apply the mathematical method of linear discriminant analysis (Demir and Ozmehmet, 2005). Using this method a discriminant function was defined in a linear form

(3) F(BFR,ITR)=0.022⋅BFR+1.61⋅BFR+1.61⋅ITR

that allowed synthesis of the desired decision rule:

(4) $$\begin{array}{ll} \text{healthy} & \text{if }F\text{(}BFR,ITR)>3.7,\\ \text{Vasospastic disorders} & \text{if }F(BFR,ITR)\leq 3.7\text{.} \end{array}$$

Substituting the experimental values of the parameters *BFR* and *ITR* into eq. (3), one can determine the presence or absence of vasospastic disorders of studied subject using decision rule (4).

**FIGURE 5:**
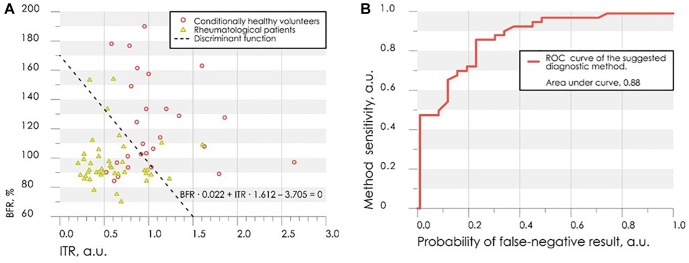
The scatter diagram of parameters *ITR* and *BFR* with the applied discriminant function **(A)** and the ROC-curve for assessing the effectiveness of the classifier **(B)**.

Figure 5A shows the scatter plot of parameters *ITR* and *BFR* with the discriminant function (3). Points of the given graph correspond to a combination of experimental values *BFR* and *ITR* for examined persons, and discriminant linear function (3) divides the feature space into two half-planes. According to the decision rule (4), the area above the discriminant straight line corresponds to the absence of vasospastic disorders in the fingers, the area below – to the presence of vasospastic disorders.

Leave-one-out cross-validation method was used for verification of the decision rule (Rao et al., 2007). Verification procedure reveals the probability of false-negative diagnosis result at the level of 0.13 (sensitivity 0.87), false-positive at 0.26 (specificity 0.74). The receiver operating characteristic curve (ROC-curve) for the suggested method of diagnosis is shown in Figure 5B. It indicates the quality of the suggested method of binary classification. The curve shows the sensitivity to specificity ratio at various values of the threshold between the domains of normal state of health and angiospastic disorders. The area under the curve is 0.88, which is indicative of a high efficiency of the classifier.

The proposed decision rule (4) allows us to offer a diagnostics method for the functional state of peripheral vessels, which can be characterized as reserve possibilities of blood flow using the LDF method, and reactivity of peripheral vessels located at a greater depth using the cutaneous thermometry method.

### Experimental Results and Discussion

The new diagnostic procedure using methods of laser Doppler flowmetry and thermometry during combined provocative factors of cold and ischemia was proposed in the study. It was suggested to use the measurements of blood microcirculation and skin temperature during the occlusion test in the thermally stabilized environment as a diagnostic approach for identification dysfunctions of the peripheral blood vasculature in PRD.

Using the experimental values of the parameters of LDF- and thermograms, a simple model for the classification of the presence or absence of vasospastic disorders in the fingers was proposed. The resulting classification model showed good results of sensitivity (0.87) and specificity (0.74).

The proposed approach of using a combination of several diagnostic technologies makes it possible to conduct a comprehensive assessment of the functional state of microcirculation vessels and larger vessels of the fingers. The combined effect of low temperature and temporary circulatory arrest provokes a manifestation of the vasospastic state. Since the vasospasm appears sporadically, this approach allows reducing the level of false–negative diagnostic results.

The results from this conducted research can be used in the development of multi-functional non-invasive diagnostic systems for the diagnosis and prevention of diseases associated with changes in the functional state of peripheral vessels.

## Cold Pressor Test in Detection of Disorders in the Microcirculatory Bed of Upper Limbs

### Materials and Methods

To evaluate the combined use functionality of the laser Doppler flowmetry (LDF), tissue reflectance oximetry (TRO), pulse oximetry (PO) methods and cold pressor test (CPT), experimental studies involved 32 HV (mean age 22 ± 2 years) and 60 PRD (mean age 55 ± 14 years) from the Rheumatology Department of the Orel Regional Clinical Hospital (Oryol, Russia).

The group of patients includes individuals primarily with the rheumatoid arthritis and systemic lupus erythematosus.

As described previously, disorders of upper limb microcirculatory bed are most commonly found as one of the forms of rheumatological profile disease pathologies. These diseases are more common in the elderly. It is thus necessary to clearly differentiate between a healthy state and one with microcirculatory bed disorders. A group of healthy young volunteers was recruited as a control to ensure an “extreme” state of good health, as they would present the lowest chance of exhibiting any undesired physiological conditions.

**FIGURE 6:**
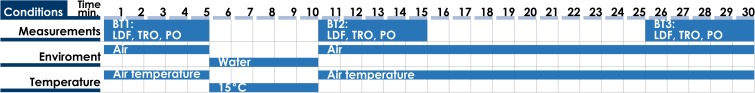
Time chart of the study protocol for the diagnostics of the microcirculatory bed of upper limbs: BT1, BT2, and BT3 – basic tests before, immediately after and 15 min after the cold exposure.

The experiment was conducted according to the study protocol described in Figure 6.

All measurements were performed in conditions of physical and mental rest 2 h after a meal. Volunteers also underwent a preliminary adaptation to room temperature 24–25°C for 15–20 min in a sitting position, with the right arm on the table at heart level. The adaptation of volunteers to standard room temperature and abidance of study protocol during all measurements reduces the influence of different factors on results of diagnosis.

**FIGURE 7:**
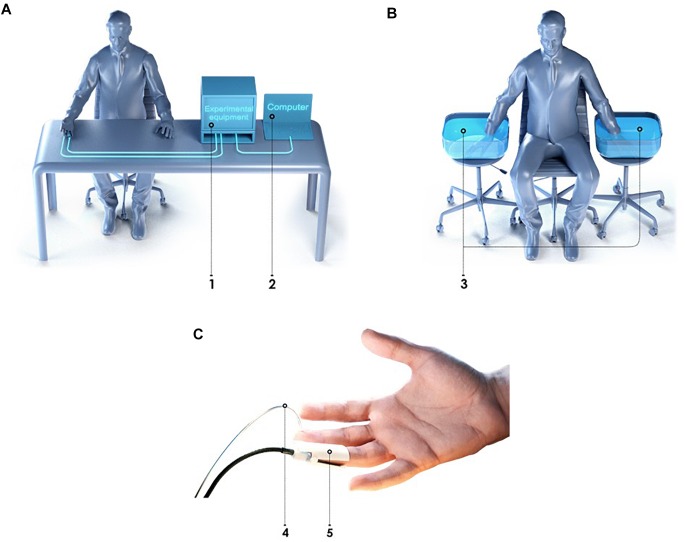
Schemes of experimental installation for the measurement of the microcirculatory bed of upper limbs **(A)**, during the cold exposure **(B)** and LDF and TRO probes location on a finger **(C)**: 1 – experimental equipment, 2 – computer, 3 – container with water, 4 – pulse oximetry sensor, 5 – LDF and TRO probe.

Experimental systems “LAKK-OP” and “LAKK-M” (SPE “LAZMA” Ltd., Russia) were applied for the measurement LDF-, TRO-, and PO-signals. These diagnostic devices utilized identical measurement channels. Temperature of water in a container during the cold exposure was controlled by a contactless digital thermometer (Sensitec NB401, Netherlands). Schemes of experimental installation during the measurement, during the cold exposure and schematic presentation of the LDF- and TRO-probe and pulse oximetry sensor positioning on a finger are showed in Figure 7.

The used TRO channel calculates the tissue oxygen saturation, which is defined as the percentage composition of oxyhaemoglobin in the sum of major fractions of hemoglobin in a tissue volume (Dunaev et al., 2014). The instrument implements the computational model based on the modified diffuse approximation of the light transfer equation (Spott et al., 1997) and utilizes the differences in the spectral characteristics of oxygenated and deoxygenated hemoglobin. The TRO measurements allow for non-invasive monitoring of microhaemodynamics and transport and utilization of oxygen within the blood microcirculation system.

Basic microcirculatory bed parameters were registered during experimental studies, providing a vast array of information. Index of blood microcirculation (*I*_m_), tissue oxygen saturation (*S_t_O_2_*) and arterial oxygen saturation (*S_à_O_2_*) were registered by proposed methods.

Wavelet analysis of the registered LDF- and TRO-signals was carried out to evaluate the regulatory mechanisms. The complex Morlet wavelet was used as the analyzing wavelet (Frick et al., 2015). The calculation of wavelet coefficients for the frequency range from 0.01 to 2 Hz was performed with a logarithmic partitioning into 50 frequency sub-bands. Global wavelet power spectra were also calculated for both study groups in each BT. Maximum amplitude of peripheral blood flow oscillations in one of the frequency bands [endothelial (*A*_e_), neurogenic (*A*_n_), myogenic (*A*_m_), respiratory (*A*_r_) and cardiac (*A*_c_)] calculated from the wavelet analysis of LDF- and TRO-signals (Goltsov et al., 2017). Based on measured parameters and results of wavelet analysis of the registered LDF- and TRO-signals using proposed approach (Makovik et al., 2017) complex parameters were calculated.

Statistical analysis of the measured and calculated parameters was performed using non-parametric criteria: the Mann–Whitney test for comparing values between groups and the Wilcoxon test for comparing values within a single group.

### Data Analysis and Diagnostic Criteria

**FIGURE 8:**
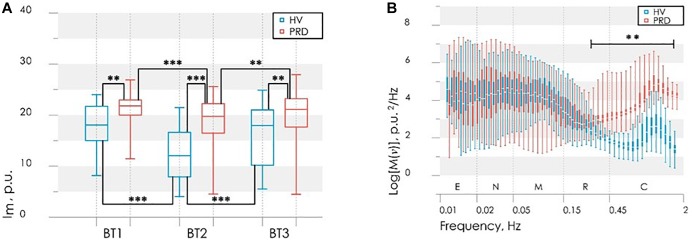
Results of the analysis of perfusion in BT1, BT2 and BT3 **(A)**; power spectral density in BT1 **(B)**. Statistically significant differences in the values with ^∗∗^*p* < 0.01, ^∗∗∗^*p* < 0.005; E, endothelial; N, neurogenic; M, myogenic; R, respiratory; C, cardiac frequency bands of blood flow oscillations.

Results of experimental studies showed that CPT evokes different reaction from the microcirculatory bed in each group. In particular, partial or complete recovery of the blood flow parameters after the CPT in some subjects was observed. As it is shown in Figure 8 higher perfusion in basal state in PRD group was observed, herewith cooling provokes the more evident response to changes of temperature in HV (Mizeva et al., 2017).

Interestingly, that analysis of averaged wavelet spectra in BT1 showed that larger energy in high frequency pulsations (above the frequency of 0.24 Hz) of the blood flow were observed in PRD than in ÍV, herewith this energy saved and after the cooling. The difference between groups in the low-frequency part of the spectra does not observe. After 20 min total restore of blood flow as far as spectral composition was observed.

As was previously stated in the works (Gutierrez et al., 2010; Yildiz, 2010) this reaction is associated with a weak damping capacity of the vascular bed due to a decrease in the elasticity of the vascular wall and increase its stiffness, and also because of morphological disturbances arising during RD (formation of megacapillaries, thinning of the capillary network).

Based on the obtained results and the differences between HV and PRD, the values of the perfusion and the maximum amplitude of LDF oscillations during CPT was used to the synthesis of the decision rule for diagnose microcirculatory disorders in RD. These parameters satisfy the principles of statistical independence, as well as the significance of the differences of their values, calculated for the PRD and HV. A discriminant function of the values of perfusion *I*_m2_ and the maximum amplitude of blood flow oscillation in cardiac frequency band *A*_c2_ for BT2 (measurement immediately after the cold exposure) was defined in the following linear form

(5) $$F(I_{m2},A_{c2})=0.12\cdot I_{m2}+1.93\cdot A_{c2}-3.25$$

**FIGURE 9:**
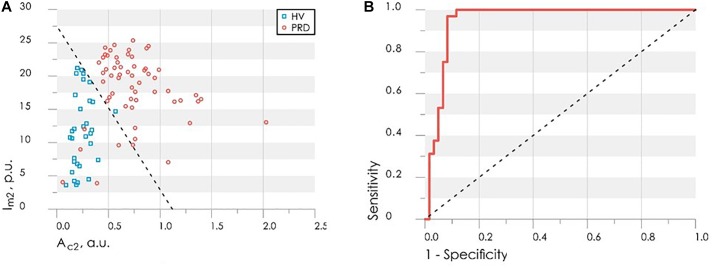
The scatter diagram of parameters *A*c2 and *I*m2 with the applied discriminant function (straight line), obtained by LDF method **(A)** and the ROC-curve (solid line) for assessing the effectiveness of the classifier **(B)** Dash line shows ROC-curve with area under curve equals of 0.5 and characterizes the unsuitability of the classification method.

Figure 9A shows the scatter plot of parameters *I*_m2_ and *M*T_2_ with the discriminant function (5). Points of the given graph correspond to a combination of experimental values *I*_m2_ and *M*T_2_ for HV and PRD, and discriminant linear function (5) divides the feature space into two half-planes. The area above the discriminant straight line corresponds to the absence of microcirculatory disorders in the fingers, the area below – to the presence of microcirculatory disorders.

Figure 9B shows the ROC curve calculated for the obtained discriminant function. Area under curve (AUC) was used to compare the quality of different classifying rules. For the synthesized decision rule AUC equals 0.92. The results demonstrate that the perfusion and amplitude of the pulse wave can act as independent markers for microcirculatory disorders in RD.

**FIGURE 10:**
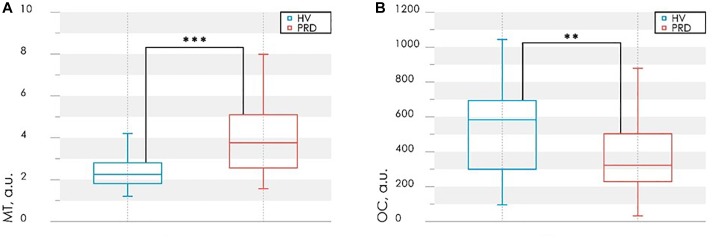
Results of the analysis of the myogenic tone, *MT* **(A)** and rate of oxygen consumption, *OC* **(B)** in BT3 (measurement 15 min after the cold exposure). Statistically significant differences in the values with ^∗∗^*p* < 0.01, ^∗∗∗^*p* < 0.005.

Additional calculated parameters have been evaluated as it described in Makovik et al. (2017). Analysis of the parameters in each group during experimental study identified differences in myogenic tone (*MT*) and rate of oxygen consumption (*OC*) in BT3 (measurement 15 min after the cold exposure) (Figure 10), namely higher level of *MT* and lower level of *OC* in PRD than in HV.

Differences in *OC* level in PRD and HV (Figure 10B) indicate the possible violations of the microvascular bed surface of the smallest arterioles and capillaries (Makovik et al., 2017). In PRD a decrease of *OC* with an increase of *MT* compared with the values for HV are observed (Figure 10A). Such result can be interpreted as a sign of reduction of oxygen diffused through the vessel walls. These processes can lead to hypoxia, edema of tissues and the appearance of necrobiotic processes at untimely diagnostics and absence of treatment.

An analysis of the possible causes of these pathological changes revealed their association with an increase in myogenic tone, as well as its combination with venous stasis. So *MT* and *OC* can be used as an additional diagnostic criteria for detection of complications associated with microvascular disturbances and their possible causes.

### Experimental Results and Discussion

Experimental studies have shown that the combined use of optical non-invasive technologies (laser Doppler flowmetry and tissue reflectance oximetry) in combination with cold pressor test has a huge diagnostic potential. The described method allows diagnosing the presence of vasospastic disorders in rheumatic diseases and identifying the possible cause of the pathological condition.

The proposed approach is based on the use of LDF and TRO methods before and after the exposure and subsequent wavelet transform of the signals. The level of the skin blood flow and its spectral properties undergo changes due to violations in the microcirculation system regulation. The mean level of the local microcirculatory blood perfusion and the amplitude of the pulse oscillations of blood flow immediately after CPT are two proposed diagnostic criteria, which were chosen for the synthesis of the decision rule. The resulting classification model provides excellent results of sensitivity (0.92) and specificity (0.97) in diagnosing microvascular disorders in rheumatic diseases (see Figure 9B).

In case of detection of microcirculatory disorders, the second stage of the proposed diagnostic method was implemented. It consists in identification of the associated complications and their possible causes with the use of additional diagnostic criteria: complex parameters of hemodynamics (myogenic tone, *MT*) and tissue respiration (rate of oxygen consumption, *OC*). The parameters are calculated based on the measured LDF and TRO parameters before and after CPT. The combination of parameter values allows assessing the presence of complications and identifying myogenic or myogenic-congestion cause of their occurrence.

The clinical trail of the method showed a considerable promise in clinical application for the functional evaluation of microcirculatory blood flow regulation and has good possibilities in the diagnosis of microvascular disorders associated with rheumatic diseases.

## Multimodal Optical Measurements Under Local Heating Test to Reveal Changes in Lower Limbs in Patients With Diabetes Mellitus

### Materials and Methods

The aim of this study was to estimate experimentally the potential of co-registering parameters of cutaneous blood flow and the intrinsic tissue fluorophore fluorescence with the object to determine the stage of lower limb complications of in patients with diabetes mellitus.

The tissue blood perfusion and the tissue fluorescence were assessed in the experimental studies by LDF and FS, respectively (Dremin et al., 2017). Parameters of the blood perfusion and the amplitude of the coenzyme NADH and FAD fluorescence signals were registered simultaneously in the same tissue volume. The single mode laser module with a wavelength of 1,064 nm was used in the laser Doppler channel of the system SPE “LAZMA” Ltd., Russia. For the fluorescence excitation, two radiation sources of 365 and 450 nm were applied. For the assessment of the local regulatory mechanisms of blood flow the local heating stimulation was selected as a provocative action on the blood microcirculation system. A special block “LAZMA-TEST” (SPE “LAZMA” Ltd., Russia) allowed us to establish and maintain the determined local temperature of the investigated area. Specialized software was used to control the unit responsible for thermal functional tests, to observe the real-time course of the experiment and to analyze the recorded parameters.

The experimental study involved 76 patients from the Endocrinology Department of the Orel Regional Clinical Hospital (Oryol, Russia). All patients had type 2 diabetes mellitus. The illness was taking its normal course in 62 patients (Diabetic group 1, mean age 54 ± 10 years). Another 14 people among the patients’ group were identified to have a more severe course of the disease (Diabetic group 2, mean age 53 ± 13 years). In each case, the degree of severity was determined based on anamnesis analysis, consultation with the attending physician and presence of trophic disorders: Diabetic group 1 consisted of all patients without trophic ulcers, when Diabetic group 2 included diabetics with the presence of visible trophic ulcers. The control group consisted of 48 healthy volunteers (mean age 46 ± 6 years).

**FIGURE 11:**
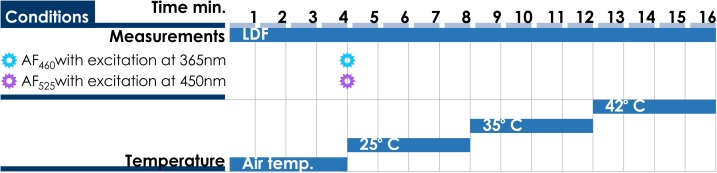
Time chart of the study protocol of multimodal optical measurements under local heating test.

The experimental study was conducted under the established protocol. Each experiment consisted of four stages. The first stage included 4 min registration of microcirculatory blood perfusion under the basal conditions and recording of a pair of fluorescence spectra at the end of the period. This was followed by a consecutive local cold (25°Ñ) and heat (35 and 42°Ñ) provocations lasting for 4 min each. The duration of one experiment was approximately 16 min. Time chart of the study protocol is shown in Figure 11.

**FIGURE 12:**
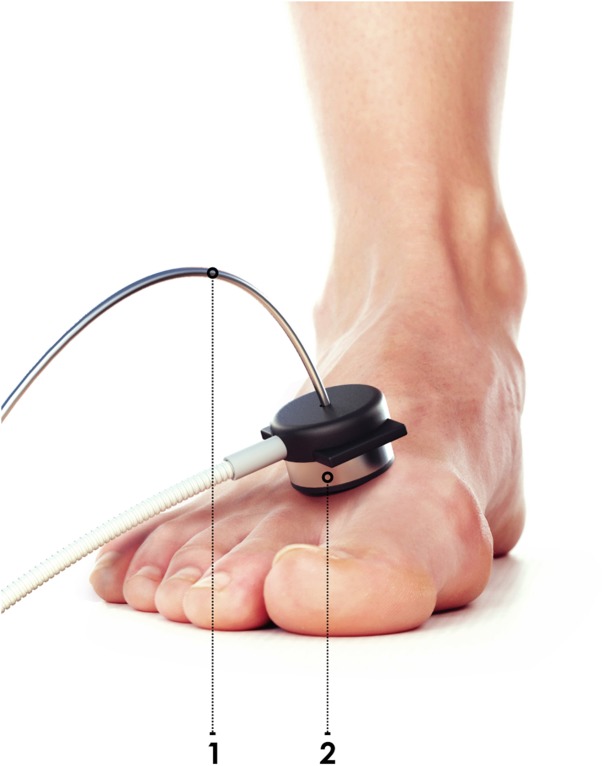
Location of the fiber optic probe (1) with Peltier thermal-pad (2).

The dorsal surface of the foot was chosen as an investigated area. The probe was secured on a plateau between the 1st and 2nd metatarsal bones (Figure 12).

Changes in blood flow of the investigated area of non-glabrous skin are mediated by the vessels themselves and by mechanisms of the sympathetic nervous system (Johnson and Proppe, 2011). The microcirculation assessment in this area may demonstrate better sensitivity to disease severity in comparison with glabrous or non-hairy skin (with arteriovenous anastomoses) where blood perfusion may fluctuate substantially (Thoresen and Walløe, 1980; Vanggaard et al., 2012; Fuchs et al., 2017). All measurements were performed in the supine position. As local pressure of the probe on skin influences strongly on the measurement results of blood perfusion and fluorescence intensity, a particular attention was paid to minimize the parameter. The volunteers were asked to refrain from food and drink 2 h before the study to exclude the influence of these factors on the change of microhemocirculation and metabolic processes. Room temperature was maintained at a steady 24–25°C. All volunteers underwent preliminary adaptation to these conditions for at least 10 min. Change of skin temperature was recorded during the study. Since skin temperature of subjects was different in the basal conditions, the studied area was pre-cooled to 25°C to unify measurements.

### Data Analysis and Diagnostic Criteria

Averaged amplitudes of fluorescence were recorded at the basal conditions. Normalized intensities of backscattered excitation radiation (*AF*_460_ at 460 ± 10 nm with excitation at 365 nm and *AF*_525_ at 525 ± 10 nm with excitation at 450 nm) were analyzed. The wavelengths were chosen to maximize the probability of detecting NADH and FAD fluorescence signals (Dunaev et al., 2015). The microcirculatory blood perfusion of the investigated tissue was recorded and averaged at each stage of the experiment. Non-parametric methods (Mann–Whitney *U*-test and Kruskal–Wallis ANOVA test) were used to confirm the reliability of differences in the results. The difference was considered significant when *p* < 0.01.

Taking into account the division of experimental data into three classes (two diabetic groups and one control), a linear discriminant analysis was used to determine the discriminant functions, which allow stating the desired decision-making rules. Thus, the obtained classifiers allow the newly appearing object to be assigned to one of the above classes by the measuring of its parameters. The quality of the discriminant analysis was assessed using the ROC-curve.

**FIGURE 13:**
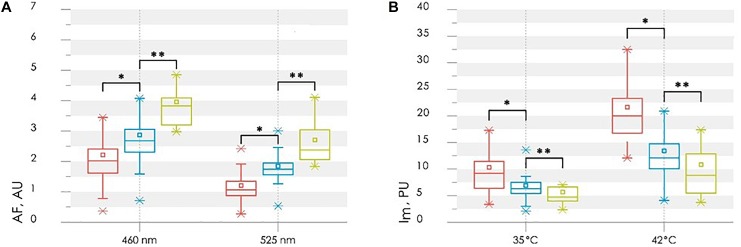
Comparison of the parameters between control (red bars), diabetic (blue bars) and diabetic with ulcers (green bars) groups: the normalized fluorescence amplitude, AF **(A)** and the average perfusion, *I*m, in the stages of heating to 35 and 42°C **(B)**. Statistically significant differences between Control group and Diabetic group 1 with ^∗^*p* < 0.01 and between Diabetic groups 1 and 2 ^∗∗^*p* < 0.01, respectively.

Experimental studies have shown that patients with diabetes mellitus have increased values of normalized fluorescence amplitudes at basal conditions and reduced perfusion response to local heating (up to 35 and 42°C). It was also noticed that these parameters in the Diabetic group 2 (patients with diabetic trophic ulcers) had significantly difference from the Diabetic group 1 and control group (Figure 13).

Statistically significant differences in parameters may be due to a number of reasons. Diabetes can cause tissue hypoxia, which leads to a violation of the aerobic respiration pathway of cells (Ditzel and Standl, 1975; Thangarajah et al., 2009; Xi et al., 2016). In this case, the mitochondrial oxidation of NADH slows down, while the glycolysis pathway of NADH formation activates. Therefore, NADH accumulation may be considered as a sign of tissue hypoxia, and its contribution to the total fluorescence signal as a marker of general oxygen deficiency in tissues.

As was mentioned earlier, AGEs are involved in the mechanisms of diabetes complications development. The rapid formation of intracellular AGEs contributes to the violation of protein function and can serve as an objective marker of glycation in tissues (Gkogkolou and Böhm, 2012). Observed in the study level of skin fluorescence is related to the determined *in vitro* standard glycation marker HbA1c. The conventional measure of glycation using HbA1c characterizes the glycation processes that occurs in a short period (around 3 months). While long-term processes in the diabetic skin are reflected by changes in AGE content. In view of the stability of AGEs and the long molecular lifetime of collagen, it might be supposed that the skin fluorescence would be applied as a measure of the total impact of hyperglycaemia in the course of life.

The basal level of perfusion does not have a statistically significant differences between the compared groups (Figure 13B). However, the proposed functional thermal tests allow identifying the microvascular disorders. Normally, the increase in perfusion during the local heating occurs mainly due to two mechanisms. As reported, skin heating to 34–35°C leads to activation of peptidergic nerve fibers by activating heat-sensitive receptors (Stephens et al., 2001). Skin heating to 42°C leads to vasodilation by releasing NO from the vascular endothelium (Minson et al., 2001; Kellogg et al., 2009). In the case of diabetes, all aspects of the microcirculatory and tissue systems, including vascular endothelial and perivascular nerve fibers, are subject to dysfunction. Therefore, the thermal test is pathogenically justified for the diagnosis of these disorders in diabetes. The reduction of perfusion growth in patients with diabetes mellitus when heated to 35°C is an objective criterion for impaired function of sensory nerve fibers, which is a component of diabetic neuropathy. The reduction of perfusion growth when heated to 42°C reflects a deficiency of endothelium-dependent vasodilation mechanisms. Differences in perfusion reaction to various stimuli can be explained by the fact that NO synthesis can be suppressed by the accumulation of AGEs in endothelial cells (Bucala et al., 1991; Stitt et al., 1997; Chakravarthy et al., 1998).

**FIGURE 14:**
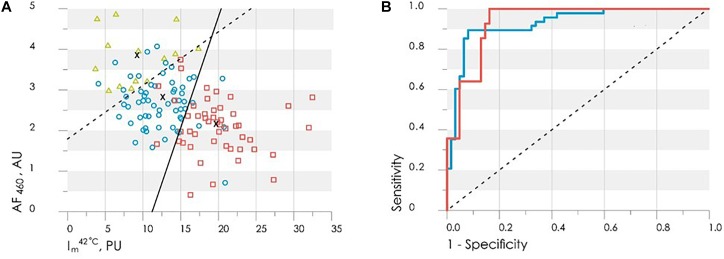
**(A)** The scatter plot of the values *I*m42°C and *AF*460 obtained by LDA method with the applied discriminant functions (6) shown by solid (D2) and dash (D1) lines. The healthy group is shown by red squares, the diabetic group – blue circles, the diabetic group with ulcers – green triangles. **(B)** The ROC-curves for assessing the effectiveness of the classifiers (6): red line is Control group vs. Diabetic group 1, the blue line is Diabetic group 1 vs. Diabetic group 2, dash line is the worst case.

For the synthesis of the decision rule, the analyzed parameters of normalized amplitudes of skin fluorescence and perfusion are proposed. The selected parameters satisfy the principles of statistical independence and the significance of the difference between their values in target groups. Discriminant function was synthesized to provide the best combination of sensitivity and specificity. The lowest level of error was obtained by combining the fluorescence intensity at the excitation wavelength of 365 nm and the average level of skin blood perfusion at 42°C. Sensitivity and specificity of the first classification rule (Control group against Diabetic group 1) were 0.92 and 0.90, respectively. Sensitivity and specificity of 0.86 and 0.85, respectively, were obtained for the second classification rule (Diabetic group 1 vs. Diabetic group 2). Figure 14A shows the scatter plot of experimental data with applied discriminant lines dividing the experimental points into three groups. Thus, the resulting diagnostic criterion, using two discriminant functions, D1 and D2, and allowing classifying the measured subject to one of three groups, can be written as:

(6) $$\begin{array}{l} D_{1}=-2.55-0.45\cdot AF_{460}+0.23\cdot I_{\text{m}}^{42{}^{\circ}\text{C}},\\ D_{2}=-1.80+1.16\cdot AF_{460}-0.18\cdot I_{\text{m}}^{42{}^{\circ}\text{C}} \end{array}$$

Figure 14A shows that the shift of the values *I_m_*^42^ (°Ñ and AF460 towards the top-left corner of the diagram characteriszes the deterioration of the patient’s condition and increasing the risk of foot ulcers development.

(°Ñ and AF460 obtained by LDA method with the applied discriminant functions (6) shown by solid (D2) and dash (D1) lines. The healthy group is shown by red squares, the diabetic group

The ROC-curves calculated for the obtained discriminant functions are shown in Figure 14B. The integral characteristic – area under curve (AUC) was used to evaluate and compare the quality of different classification rules. AUC = 0.93 for both found classification rules that means a high level of the classifiers quality.

Thus, the values of skin fluorescence and level of blood perfusion during the local heating test can be used effectively for the analysis the various stages of diabetes, from the initial stage of the disease, to the development of trophic ulcers.

### Experimental Results and Discussion

The proposed method, based on the use of optical non-invasive diagnostic methods in combination with local heat test, shows the high sensitivity and can be an effective marker of metabolic changes in diabetes mellitus. Both approaches (laser Doppler flowmetry and fluorescence spectroscopy) individually and in combination have excellent diagnostic potential as they can help identify patients at risk of future problems in their lower limbs. The obtained experimental results confirm the prospects of the combined use of these optical non-invasive methods in the detection of biological tissue disorders in type 2 diabetes. In addition, the method has a good potential in the field of evaluation of therapeutic interventions aimed at preventing and reducing the progression of diabetic complications, as well as to minimize their negative consequences.

Expanding the number of clinical trials and improving the methodological support of optical non-invasive diagnostics will allow introducing these methods into clinical practice of the attending physician. The use of this methods of diagnosis of the diabetes complications, which is easily implemented, non-invasive convenient and safe for the patient, will make a significant contribution to the fight against diabetes.

One promising area for further development in this area is the differentiation and evaluation of the contributions of the AGEs, NADH, and FAD to the resulting fluorescence signal. This would allow carrying out studies to identify possible pathways of biological tissue disturbance.

## Conclusion

The use of optical non-invasive diagnostic methods has a great potential for the detection of concomitant microcirculation disorders in patients with rheumatic diseases and diabetes. In this review, it was shown that the use of laser Doppler flowmetry, optical tissue oximetry and fluorescence spectroscopy together or separately may have important diagnostic value for the detection of violations, assessment of their severity, as well as for the analysis of the effectiveness of the therapy. The joint application of the considered technologies with the methods of machine learning (discriminant analysis) can be used as additional diagnostic criteria in the field of rheumatology and endocrinology.

Thus, the introduction of optical methods for the assessment of peripheral hemodynamics and metabolic processes in rheumatic diseases and diabetes makes it possible to early preclinical diagnosis of microcirculatory and metabolic disorders, contributing to the improvement of the effectiveness and quality of treatment.

The perspective direction of the development of the optical non-invasive diagnostics method is their realization in the form of the wearable compact wireless devices suitable for long-term monitoring of microcirculation and metabolism parameters accomplished with the development of methodology of their application. The use of compact wearable devices will allow diagnosis of arterio-venular anastomoses and investigation of the compensatory mechanisms and synchronization of blood flow oscillations during functional tests.

## Ethics Statement

These studies were carried out in accordance with the Declaration of Helsinki principles and approved by the Ethics Committee of the Orel State University. All patients participated in the trials have given the full consent on measurements in written and been informed of their right to discontinue participation at any time.

## Author Contributions

AD, SS, and ER designed the study. AZ, VD, IM, and EZ collected and analyzed researched data and drafted the manuscript. AG contributed to the data analysis and preparation of a final version of the article. All authors contributed to the discussion and reviewed and edited manusript.

## Conflict of Interest Statement

The authors declare that the research was conducted in the absence of any commercial or financial relationships that could be construed as a potential conflict of interest.
